# Transesophageal echocardiography (TEE)-guided transvenous pacing (TVP) in emergency department

**DOI:** 10.1186/s13089-023-00332-7

**Published:** 2023-08-21

**Authors:** Osman Adi, Chan Pei Fong, Madeleine Kho Huei Tze, Azma Haryaty Ahmad, Nova Panebianco, Asri Ranga

**Affiliations:** 1 Resuscitation & Emergency Critical Care Unit, Trauma & Emergency Department, Hospital Raja Permaisuri Bainun, Ipoh, Perak Malaysia; 2 Trauma & Emergency Department, Sarawak General Hospital, Kuching, Sarawak Malaysia; 3 Division of Emergency Ultrasound, Department of Emergency Medicine, Hospital of the University of Pennsylvania, Philadelphia, PA USA; 4 Department of Cardiology, Serdang Hospital, Kajang, Selangor Malaysia

## Abstract

**Background:**

Placement of a temporary pacemaker is a vital skill in the emergency setting in patients that present with life-threatening bradycardia. Transvenous pacing is the definitive method of stabilizing the arrhythmia compared to transcutaneous pacing, as it provides more comfort and better control of heart rate, until the insertion of a permanent pacemaker.

**Case report:**

In this case report, we describe the steps using TEE to guide the insertion of transvenous pacer at the emergency department. Traditionally, the process of floating a transvenous pacer wire is performed “blindly” using landmarks and a monitoring ECG finding for capture, or under transthoracic echocardiography (TTE) ultrasound guidance. The blind procedure is associated with higher rate of failure and complications. While guidance using TTE is associated with higher success rates and fewer complications, inadequate imaging of the right side of the heart may limit the utility of this imaging modality. The use of transesophageal echocardiography (TEE) by emergency medicine and critical care physicians has gained traction in recent years due to its clear images and lack of interference with procedures being performed on the chest. In this article, we describe a protocol using TEE to guide the insertion of transvenous pacer through a case illustration.

**Supplementary Information:**

The online version contains supplementary material available at 10.1186/s13089-023-00332-7.

## Introduction

Temporary cardiac pacing is a life-saving procedure for hemodynamically unstable patients with bradycardia in the emergency department (ED) [1]. Indications for emergent cardiac pacing include unstable bradycardias due to degenerative conduction system disease, acute coronary syndrome complicated by bradycardia, among other reasons (Table 1).

**Table 1:** Indications for emergency cardiac pacing

Indications for emergency cardiac pacing	
1	Degenerative conduction system disease
2	Acute coronary syndromes complicated by bradycardia, AV block, or conduction system injury
3	Drug overdose
4	Overdrive pacing for ventricular or supraventricular tachycardia

Transcutaneous pacing is usually employed initially as a temporizing measure, pending placement of a permanent pacemaker in the setting of symptomatic bradycardia [2]. However, with prolonged transcutaneous pacing, capture needs to be evaluated regularly as pacing thresholds may increase. Pain resulting from transcutaneous pacing is also a common occurrence, requiring generous administration of analgesia and sedation. Periodic skin evaluation with electrode repositioning is also recommended to minimize occurrence of serious skin burns. Hence, when prolonged temporary pacing is anticipated, transvenous pacing should be considered.

Transvenous pacing, is a procedure that involves placing a catheter-based electrode into the right side of the heart through a central venous access [3]. Traditionally, fluoroscopy guidance is routinely used for placement of temporary pacing wires. However, fluoroscopy is costly, not available in the emergency department and exposes the patient to ionizing radiation. Blind, ECG-guided and transthoracic echocardiography-guided methods for transvenous pacing have been described [4]. However, transthoracic echocardiography may not provide adequate imaging of the right side of the heart, especially in the obese and those with pulmonary emphysema, sternotomy scar, and thoracic deformity.

In this case report, we describe the steps using TEE to guide the insertion of transvenous pacer at the emergency department.

## Case report

A 61-year-old Malay man with a history of hypertension and ischemic heart disease presented to the emergency department with acute onset right hemiparesis. His vital signs on arrival revealed a blood pressure 134/87, heart rate (HR) of 75 beats per minute, respiratory rate of 20 breaths per minute and oxygen saturation of 100% on room air. His Glasgow Coma Scale was E1V2M5 and his pupils were 2 mm reactive bilaterally. He was intubated for airway protection. Computed tomography (CT) of the brain revealed a left middle meningeal artery infarct.

On second day in the observation bay, the patient developed bradycardia with a HR of 30 beats per min and hypotension that did not respond to atropine. The ECG showed a complete heart block. A repeat CT brain did not reveal any new changes. His full blood count, renal profile, liver profile, serial troponin and capillary blood sugar were within normal limits. Cardiology was consulted and the ED team prepared to pace the patient.

**Fig. 1:**
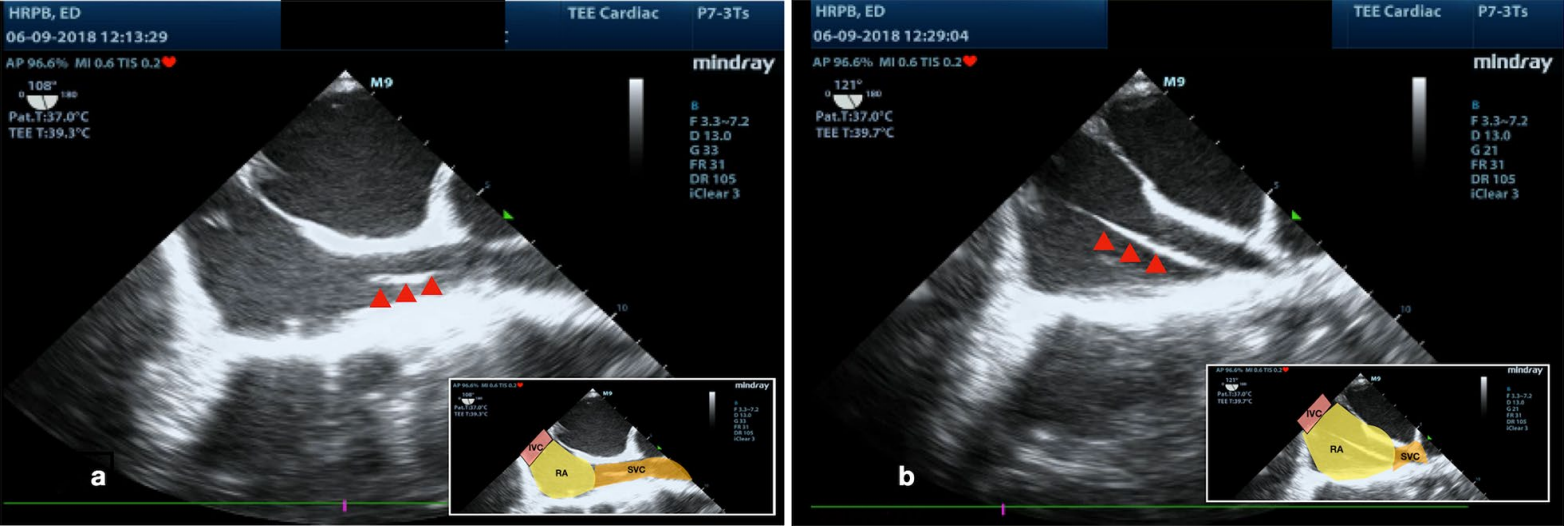
Pacing wire (red arrow head) passing through from the superior vena cava into the right atrium. RA -Right Atrium , IVC- Inferior Vena Cava , SVC -Superior Vena Cava

**Fig. 2:**
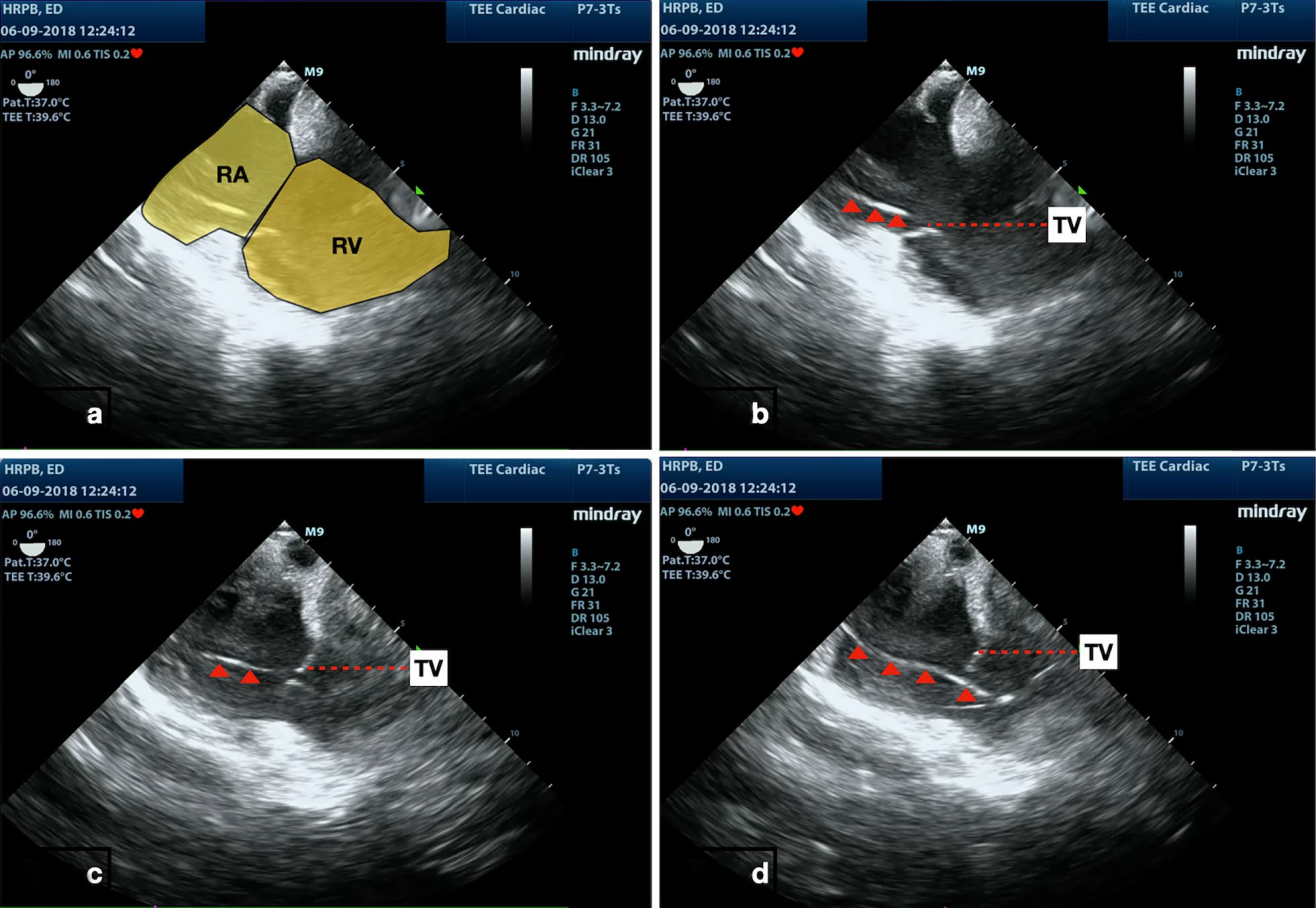
Pacing wire ( red arrow head) passing through from the tricuspid valve into right ventricle. RA -Right Atrium , RV -Right Ventricle , TV -Tricuspid Valve

**Fig. 3:**
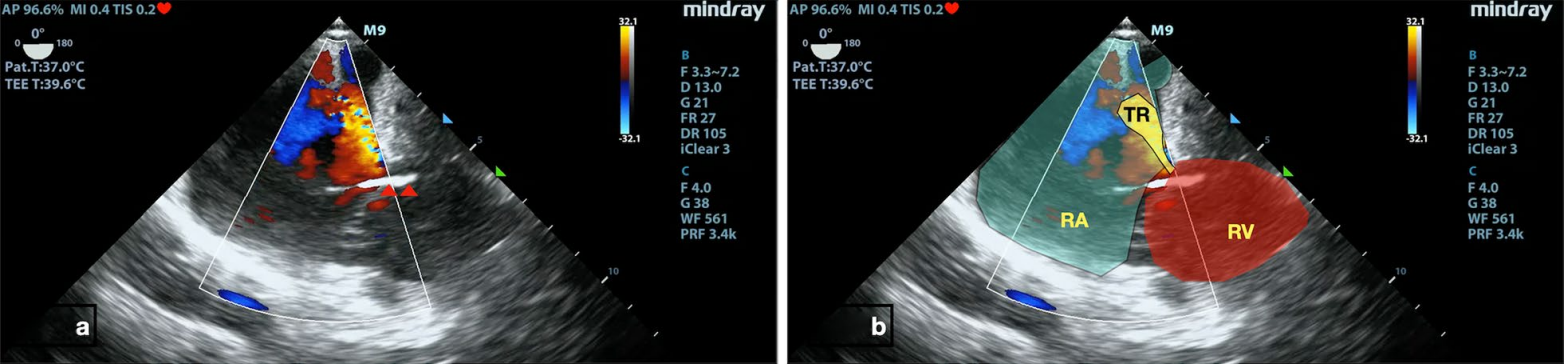
Pacing wire (red arrow head) within the right ventricle. RA -Right Atrium , RV -Right Ventricle , TR -Tricuspid Regurgitation

**Fig. 4:**
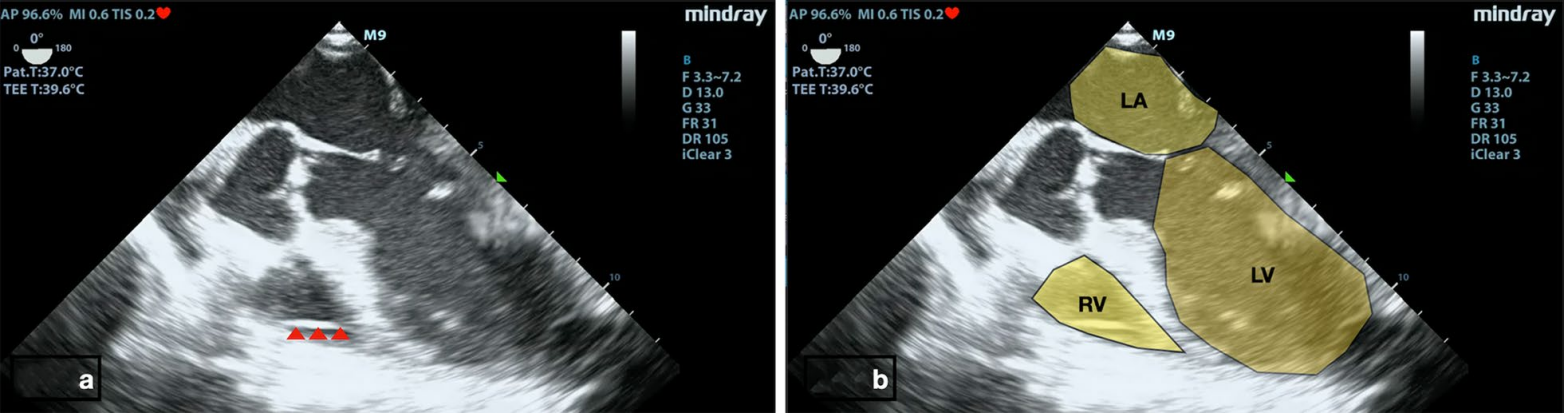
Tricuspid regurgitation jet, a sign that the pacing wire (red arrow head) has passed through the tricuspid valve to right ventricle . RA - Right Atrium , LA -Left Atrium , LV -Left Ventricle

Transvenous pacing was performed at the emergency department via right internal jugular central venous access. The TTE window was poor, thus TEE was performed to assist with real-time guidance of the pacemaker wire into the right ventricle (Table 2 and Figs. 1, 2, 3, 4). Electrical capture was obtained with a HR 80 beats per minute, sensitivity of 2 mV and an output of 5MV. Post procedure, his blood pressure stabilized to 126/71.

**Table 2:** Suggested protocol for TEE-guided insertion of TVP

Steps	Level and acquisition	Protocol for TEE-guided insertion of TVP
**Step 1**: Preparation		Place the patient on the monitor, continue transcutaneous pacing. Check the box and batteries
**Step 2**: CVC Cannulation		Obtain central venous access, preferably the right internal jugular vein
**Step 3**: TEE Probe insertion	**Mid esophageal level, Bicaval view** Transducer Angle: ~ 90–110º	Advance the TEE transducer to bicaval view
**Step 4**: Placement of Pacing Wire		Thread the transvenous pacing wire through the central venous line, inflate the balloon at 20 cm, continue advancing the wire until it is visualized in the superior vena cava and then into the right atrium (Fig. 1, Additional file 1: Video S1)
**Step 5**: Visualization of wire in the right heart	**Mid esophageal level-RV inflow–outflow** view Transducer Angle: ~ 50–70º **Refer to**^a^	Reduce the transducer angle to visualize the RV inflow–outflow view (Fig. 2, Additional file 2: Video S2)
**Step 6**: Placement in right ventricle		Advanced the transvenous pacing wire through tricuspid valve into the right ventricle (Fig. 3, Additional file 2: Video S2)
**Step 7**: Color Doppler Test		Apply color Doppler over the tricuspid valve. Tricuspid regurgitation is expected if the wire has passed through the valve (Fig. 3)
**Step 8**: Confirmation of the wire tip location	**Mid esophageal level, 4 or 5 chamber view** Transducer Angle: ~ 0–10º **Refer to**^a^	Guide the transvenous pacing wire into position with the tip in the apical right ventricular endocardium (Fig. 4, Additional file 3: Video S3). Confirm the adequate contact of the tip by advancing the probe to visualize the 4 or 5- chamber view
**Step 9**: Perform Electrical capture		Connect the pacing wire to pacing box. Set to demand. Turn rate to 30 bpm greater than intrinsic rate. Set output to 4 mA. Confirmation electrical with cardiac monitoring & ECG. Reduce the amperage until the power threshold is obtained and then double it
**Step 10**: Checking complications		Secure the transvenous catheter and screen for post procedural complications such as pneumothorax, perforation resulting in pericardial effusion

^a^Additional views: in situations where we are unable to provide more detailed information about the right heart chambers and pacemaker wire placement

ME level (with omniplane of 45°) at short-axis view of the aortic valve (Additional file 4: Video S4)

Deep transgastric view at the level of peak aortic valve, with slight clockwise rotation (transgastric longitudinal section) (Additional file 5: Video S5)

The patient was subsequently admitted to the cardiac care unit, a permanent pacemaker was implanted, and he was discharged well after 1 week.

## Discussion

Temporary transvenous pacemaker placement (TVP) is an emergency procedure that is within the scope of practice of a trained emergency medicine physician [4–7] for patients with unstable bradyarrhythmias. Due to the increasing use and benefits of the transesophageal probe, the intensivist and emergency department physician must be trained to handle this probe smoothly. In this article, we describe the process of performing TVP placement under TEE guidance using a systematic protocolized approach.

There is growing evidence demonstrating the feasibility, safety and clinical value of TEE performed by emergency physicians in the acute care setting [8, 9]. Focused TEE in the ED had been described in the management of cardiac arrest [10, 11] undifferentiated shock [12] and trauma [13, 14]. In contrast to the comprehensive TEE protocol with 28 views performed by the cardiologists, focused TEE is limited to few important views that are essential for resuscitation, namely: mid-esophageal (ME) 4 chamber view, ME 2 chamber view, ME long-axis (LAX) view, ME bicaval view, ME RV inflow–outflow view, deep transgastric (TG) mid papillary short-axis view, deep transgastric (DTG) 5 chamber view, and 4 aortic views.

For TEE-guided transvenous pacing, we recommend using the ME bicaval view, ME RV inflow–outflow view, and ME 4-chamber views. In situations where we are unable to provide more detailed information about the right heart chambers and pacemaker wire placement, we suggest using additional alternative views, such as the ME level (with omniplane of 45°) at short-axis view of the aortic valve, deep transgastric view at peak AVF, and deep transgastric view at the level of the papillary muscle.

In our department, this procedure is performed in intubated and mechanically ventilated patient by emergency physicians with training in critical care and emergency ultrasound who are skilled in performing central venous access under transthoracic ultrasound guidance and focused TEE. The emergency physicians involved need to undergo focused TEE simulation training on a manikin before performing the procedure on patients under supervision [15]. There are a few contraindications to transesophageal echocardiography, namely, esophageal injury or stricture, and lack of definitive airway. Limitations of TEE also include inability to pass the TEE into the esophagus, and presence of excessive air in the esophagus which may obscure the view obtained via TEE [16].

The technique of using TTE guidance for TVP placement is well described in the literature [17]. Lerner et al. first described TVP placement under TEE guidance in the emergency department in 2019 noting significant advantages including improved visualization of right-sided cardiac structures and lack of interference from pacer pads [18]. In addition to guidance in placement, ultrasound may be useful in the assessment of loss of capture and if the patient’s condition deteriorates. In this publication, we expand on existing knowledge by providing a clear and concise 10-step protocol that describes the use of TEE for TVP placement. As the adoption of TEE for procedures and clinical decision-making increases in emergency medicine practice, this protocol may serve as an invaluable reference (Table 2).

Complications associated with TVP used to be common, affecting 1 in 6 patients [19]. However, the incidence of complications associated with this procedure have decreased with the addition of imaging such as fluoroscopy and ultrasound. In an analysis of more than 360,000 patients at the United States, it was found that TVP was a relatively safe procedure with 0.6% risk of pericardial tamponade, 0.9% risk of pneumothorax and 2.4% risk of non-pericardial bleeding [20]. Despite the theoretical benefit of TEE-guided TVP, larger scale studies are needed to determine the feasibility, safety, and efficacy of this procedure in critically ill patients in the emergency department.

## Conclusion

Transesophageal echocardiography-guided transvenous pacer wire placement is an alternative to transthoracic echocardiography and fluoroscopy guidance during the resuscitation of a critically ill patient. As the adoption of TEE for procedures and clinical decision-making increases in emergency medicine practice, a 10-step protocol describing the procedure may serve as an invaluable reference.

### Supplementary Information

**Additional file 1.** Video S1 -Pacing wire passing through from the superior vena cava into the right atrium.

**Additional file 2.** Video S2-Pacing wire passing through from the tricuspid valve into right ventricle

**Additional file 3.** Video S3 -Pacing wire within the right ventricle.

**Additional file 4.** Video S4 -ME level (with omniplane of 45-degree) at short-axis view of the aortic valve.

**Additional file 5.** Video S5 - Deep transgastric view at the level of peak aortic valve, with slight clockwise rotation (transgastric longitudinal section).

## Data Availability

The material available from the corresponding author on reasonable request.

## Declarations

I declare that this manuscript which depicts the suggested protocol using TEE in transvenous pacing placement. Contributions from respective authors have been explicitly mentioned in the respective segment. This work has not been submitted to any other publication for publishing.
